# Some, but not all, patients experience full symptom resolution and a positive rehabilitation process after ACL reconstruction: an interview study

**DOI:** 10.1007/s00167-022-07271-1

**Published:** 2022-12-09

**Authors:** Ramana Piussi, Cajsa Magnusson, Sara Andersson, Kaisa Mannerkorpi, Roland Thomeé, Kristian Samuelsson, Eric Hamrin Senorski

**Affiliations:** 1 Sportrehab, Sport Medicine Clinic, Gothenburg, Sweden; 2 Sahlgrenska Sports Medicine Center, Gothenburg, Sweden; 3 Unit of Physiotherapy, Department of Health and Rehabilitation, Institute of Neuroscience and Physiology, Sahlgrenska Academy, University of Gothenburg, Gothenburg, Sweden; 4 Department of Orthopaedics, Institute of Clinical Sciences, The Sahlgrenska Academy, University of Gothenburg, Gothenburg, Sweden

**Keywords:** Anterior cruciate ligament, ACL, Cope, Content analysis, Rehabilitation, Injury

## Abstract

**Purpose:**

To gain a deeper understanding of patients’ experiences over 5 years after anterior cruciate ligament (ACL) reconstruction.

**Methods:**

Seventeen semi-structured interviews were performed with patients treated with ACL reconstruction at least 5 years earlier without a second knee injury. Interviews were transcribed and analyzed using qualitative content analysis according to methods described by Graneheim and Lundman.

**Results:**

Patients’ long-term experiences after an ACL reconstruction were summarized as: “to cope or not to cope, that is the question”, and five main categories: (1) Adapting life after knee symptom: the past will not come back; (2) An arduous and demanding rehabilitation: sailing against the wind; (3) Accepting what cannot be changed: biting the bullet; (4) Being satisfied with results: end of a chapter; (5) Apprehensively peregrinating on an unknown road.

**Conclusions:**

More than 5 years after ACL reconstruction, patients can experience full symptom resolution and the ACL injury process as positive, or experience persistent symptoms and are forced to accept negative life-changing choices due to the injury.

**Level of evidence:**

IV.

**Supplementary Information:**

The online version contains supplementary material available at 10.1007/s00167-022-07271-1.

## Introduction

Qualitative studies on patients’ experiences after anterior cruciate ligament (ACL) reconstruction have reported that patients express feelings of uncertainty regarding whether they will fully recover [6], a lack of confidence toward the possibilities of future sports participation, and that patients perceive and report psychological barriers as greater than physical barriers during rehabilitation [4, 5, 16, 21]. Patients report that, when about to return to pre-injury physical activity after an ACL reconstruction, a primary reason for not resuming their pre-injury physical activity is fear of re-injury [13]. Knowledge on the experiences of rehabilitation in patients after an ACL reconstruction primarily focus on the rehabilitation period and the return to pre-injury physical activity, leaving a knowledge gap about the mid and long term experiences of patients after an ACL reconstruction. Up to 55% of patients treated with ACL reconstruction return to competitive sports, and 81% to any sports [2]. Consequently, there are patients who do not resume their pre-injury level of physical activity after an ACL reconstruction. Qualitative evidence allows patients to share their perception of a certain event, consenting the researcher to build a different understanding of one of the pillars of evidence-based medicine: patients’ preferences—forming evidence-based medicine alongside the best available evidence and clinical experience [3]. Qualitative research can enhance the understanding of factors that negatively affect life after an ACL injury, provide information to guide management strategies, and improve long-term recovery following ACL reconstruction.

The purpose of this study was to gain a deeper understanding of patients’ experiences more than 5 years after ACL reconstruction.

## Materials and methods

Written consent was obtained from all patients; ethical approval was obtained from the Swedish Ethical Review Authority (DNR: 2020-02501).

This study was conducted using data collected through individual semi-structured interviews. The data were analyzed using qualitative content analysis with an inductive approach, based on the methods described by Graneheim and Lundman [9, 10]. The Consolidated criteria for Reporting Qualitative research (COREQ) [20] checklist was used.

Patients participating in the present study were recruited from an ongoing rehabilitation outcome registry called Project ACL [15]. Project ACL was established in 2014. Through Project ACL’s database, eligible patients with one ACL injury, aged 18–60 years, who had undergone ACL reconstruction for a minimum of 5 years before April 01, 2021, and no subsequent knee injury were selected. A strategic selection of patients, based on patient sex and age, was then contacted by telephone by the first author (RP) of the study, informed about the study, and asked whether they were interested in participating. A total of 33 patients were contacted, of whom 16 were not interested in participating. Upon positive response, a meeting was scheduled.

A goal for a minimum of 12 patients was set based on recommendations for data saturation in interview studies [11]. In addition, saturation was continuously assessed, and data collection stopped when no further codes or subcategories emerged from the analysis.

To warrant reflexivity, that is, the ongoing process between the research data and the researchers analyzing the data with qualitative research, the first (RP, MSc) and senior (EHS, PhD) authors, both males, are experienced physical therapists (PTs) with 6–10 years of experience from working in a sports rehabilitation setting. In terms of other authors, SA and CM are two female PTs who have long been interested in sports-related injuries and rehabilitation after an ACL injury. The other three authors (RT, KS, and KM) are professors, and made important contributions to the discussion of the study ideas, and the writing of the manuscript. One is a senior female PT (KM), working as a researcher at the local university, with extensive experience in qualitative research. The fifth author (RT) is a retired senior PT (male) still active in the research field, with more than 40 years of experience in clinical practice and the research field, and the sixth author (KS) is a male orthopedic surgeon and researcher working primarily with patients with ACL injuries.

### Data collection

An interview guide was created by the first (RP), fourth (KM), and the senior (EHS) authors through discussions and screening of the literature on the subject. Appendix “A” presents the interview guidelines.

Data were collected via recorded interviews between April 28, 2021 and June 7, 2021 with 17 participants. via ZOOM, web-based application. Fifteen interviews were performed by the first author (RP) and one each by the second (CM) and third author (SA). This choice was made, because the first author, RP, had a personal connection with one of the participants. There were no other relationships between the study authors and the included participants. Participants were not aware of the personal goals or the researchers’ reasons for conducting the research. The interviews were transcribed verbatim by the first (RP), second (CM), and third authors (SA) of the study. Transcripts were not sent to the participants for corrections or comments. Table 1 presents the demographic information of the patients.

**Table 1:** Demographics for the 17 included participants

Sex, male (%)	7 (41%)
Age, years; mean; median; range	30.9; 26; 18–58
Time between injury and reconstruction days; mean; median; range	303.6; 187; 50–1385
Graft choice, hamstring (%)	17 (100%)

Interviews lasted between 14 and 30 min, for a total time of 337 min.

### Data analysis

Data were analyzed using qualitative content analysis with an inductive approach, based on the methods described by Graneheim and Lundman [9, 10]. The first (RP), second (SA), third (CM), and senior (EHS) authors were responsible for the data analysis process, which was carried out in Microsoft Excel (Microsoft Corporation, 2016).

To obtain a general understanding of the data, the transcripts were first read thoroughly several times. In the second step, meaning units were extracted and grouped into condensed meaning units. Condensed meaning units were abstracted and coded. Codes addressing similar categories were grouped into sub-categories, which were then grouped into main categories. Lastly, main categories were grouped into a theme. During the process of abstraction, coding, and categorization of codes, the interview transcripts were continually read to ensure that the data were appropriately understood in relation to the context. The analysis was performed individually by three authors (RP, CM, SA) and then triangulated in discussion with the senior author (EHS). Any divergence between the authors was resolved by discussion with the fourth author (KM). After grouping of categories, the transcripts were read again, and categories were validated against the transcripts to ensure that data were not missed, misinterpreted, or erroneously included. To increase transparency in qualitative research, an example of codes, grouping in sub-categories, and main categories is presented in Table 2.

**Table 2:** Examples of the analysis process from codes to main categories

Codes	Sub-category	Main category
Learned to live with my injury	To accept the situation	Biting the bullet: accepting what cannot be changed
Try not to think of the injury		
Want to put this journey behind		
Got used to live with an injured knee		
Life (after treatment) is not as I imagined	Unsatisfied with the outcome: something was missing	
The injury cannot be undone		
Not nice to live with an ACL injured knee		
Sorrowing not to be able to play		
Rehabilitation proceeded constantly forward	Satisfied with performance, surgery and results	End of a chapter: being satisfied with results
I had a great physiotherapist		
I was dedicated to rehabilitation		
Knee responded well to rehabilitation		
Do not think of my knee	The knee is not evocative of symptoms	
Can trust my knee		
No consequences in everyday life		
I am not affected by my knee		

## Results

**Fig. 1:**
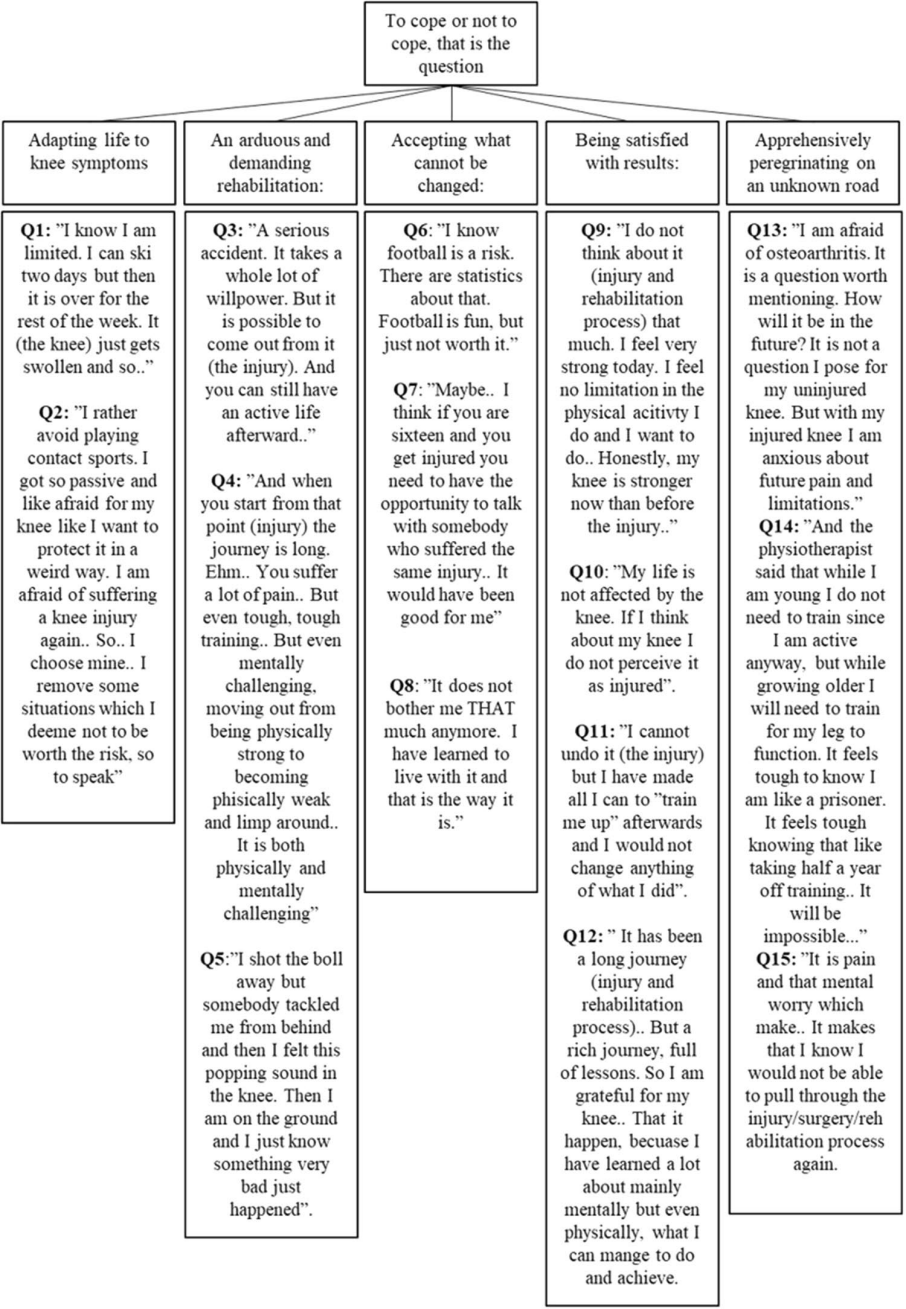
Main theme and five main categories, with quotes from interviews. Each quote is taken from a sub-category. *Q* quote

One theme, supported by five main categories, was derived from the data collected during the analysis. Table 3 presents an overview of the main themes, categories, and sub-categories. Figure 1 presents a summary of main categories and quotes from interviews.

**Table 3:** Main theme, main categories, and sub-categories, presented in the mentioned order

“To cope or not to cope, that is the question”				
Adapting life after knee symptoms: the past will not come back	An arduous and demanding rehabilitation: sailing against the wind	Accepting what cannot be changed: biting the bullet	Being satisfied with results: end of a chapter	Apprehensively peregrinating on an unknown road:
Presence of symptoms when performing knee demanding activities	Tough and long diagnosis process	Dare to return to sport?	Active with no limitations	Uncertainty about the future and osteoarthritis
	Experiences of rehabilitation	Unsatisfied because something was missing	The knee is not evocative of symptoms	Being afraid for the knee and for what might happen
Changing sport	Thoughts and feelings related to injury	To accept the situation	Satisfied with performance, surgery, and results	No wish for re-injury
			Positive lessons learned

Patients’ experiences more than 5 years after ACL reconstruction were summarized in one theme: “to cope or not to cope, that is the question”. Articulating two opposites, patients after an ACL injury can experience resolution of symptoms, return to sports and performance, and be active without limitations, or, on the contrary, need to adapt life with respect to knee symptoms, being unsatisfied with their knee outcome, but needing to accept the situation. Looking at the future, patients expressed uncertainty.

### Main category: “Adapting life after knee symptoms: the past will not come back”

With respect to the time that had passed since ACL injury and surgery, patients experienced that life in the context of their knee was characterized by symptoms and giving up on participating in their sport had to be seen as the new “normal”, and therefore, the past would not come back.

*Presence of symptoms during knee-demanding activities*: the presence of knee symptoms such as pain, perceived instability, or stiffness when performing knee-demanding physical activities, such as running or quick pivoting movements, was mentioned by the patients. The presence of knee symptoms limited certain movements, such as kneeling or deep squatting, and led to feelings of impaired knee-related self-efficacy.

*Changing sport*: when suffering an ACL injury, patients were taken away from their sports for a long period. Upon returning to sport, hindrances in reaching the pre-injury level of performance were seen as major difficulties. Consequently, the inability to perform at pre-injury levels of sport or physical activity at times led to the end of sporting careers, and sometimes, to the change of sport. The perceived risk of sustaining a second severe knee injury was expressed as a hindrance to participate in sporting activity. To hopefully not have future knee problems, some patients chose an inactive lifestyle and some chose to change sports in favor of one with less demands on the knee. [Quotes #1, #2 (see Fig. 1)].

### Main category: “An arduous and demanding rehabilitation: sailing against the wind”

The overall patients’ experience was that rehabilitation was very time-consuming and tough, both physically and mentally, and that it was, at times, difficult for patients to find motivation.

*Tough and long diagnosis process*: the time between injury and diagnosis was perceived as long and frustrating by patients. Patients expected the healthcare system to be able to take care of their problems, and therefore, difficulty in meeting competent healthcare providers who could diagnose an ACL injury was mentioned as a factor that increased frustration, as well as a disappointment toward the healthcare system. The time between diagnosis and surgery provided patients the opportunity to prepare both physically and mentally for surgery, which was experienced as positive, since patients reported having the possibility to study what to expect, and to be able to prepare for surgery by strengthening the body as much as possible.

*Experiences of rehabilitation*: patients described rehabilitation as non-linear, that is, proceeding without hindrances and being smooth sometimes, but at other times, filled with setbacks, such as pain, discomfort, or stiffness in the knee joint. For some patients, rehabilitation was considered fun and a high motivation for training with a physiotherapist was expressed. In contrast, other patients perceived rehabilitation as demanding, especially during periods perceived as boring or physically challenging. As time from injury, diagnosis, and surgery passed, rehabilitation was described as tough, both physically and mentally, but deemed necessary by the patients.

*Thoughts and feelings related to injury*: as patients who suffer an ACL injury are usually active in sports, patients are aware of what an ACL injury is. Therefore, upon suffering the injury, many patients strongly suspected that the injury was to their ACL. The early period after the injury was followed by thoughts of catastrophes, stress, and feelings of uncertainty among patients. The patients reported regretting the injury and wishing it never occurred. Furthermore, patients expressed a sense of sorrow related to the life changes that the ACL injury imposed, since changes were imposed, and as patients felt nothing could be done to alter the imposed changes, feelings of sadness and despair were expressed. [Quotes #3, #4, and #5 (see Fig. 1)].

### Main category “Accepting what cannot be changed: biting the bullet”

To move on with their lives, patients expressed a need to accept their situation, regardless of how difficult or sad it was.

*Dare to return to sport?:* there was a lack of courage to attempt a return to sports in patients due to fear of re-injury and the fear of having to go through the surgery/rehabilitation process once more, which inferred a sense of sadness. Due to the injury process, some patients reported feelings of sympathy and a sense of pity for other individuals who suffered an ACL injury.

*Unsatisfied because something was missing*: since rehabilitation did not provide the desired outcomes, some patients who gave up their sports career or were unsatisfied with knee-related function and quality of life and felt that something was missing and expressed a desire to make different treatment choices. Such changes in treatment choice included a preference for a different graft as well as training harder during rehabilitation.

Furthermore, some patients wished to seek support from others with the same injury to cope with physical and psychological demands, but not all patients who wished support could find others with the same injury.

*To accept the situation*: despite not achieving their desired outcomes, some patients learned to cope with the consequences of the injury and to live with the limitations imposed by their injured knee and accepted their situations. There was a desire to leave the ACL injury and the subsequent process behind them and move on with life, learning to live with the injured knee. [Quotes #6, #7, #8 (see Fig. 1)].

### Main category “Being satisfied with results: end of a chapter”

*Active with no limitations*: some patients successfully returned to the pre-injury level of sports and felt joy to be able to participate without restrictions and limitations. The ability to perform their sport without limitations or knee symptoms allowed for continued investment toward their sporting career without fear of re-injury.

*The knee is not evocative of symptoms*: as knee functions and knee-related quality of life were satisfactory, some patients were not constantly reminded of their knee injury.

*Satisfied with performance, surgery, and results*: patients expressed satisfaction with the outcomes of the surgery/rehabilitation process. Alongside satisfaction with treatment outcomes, there was also satisfaction with the patients’ own efforts and contributions, leading to recovery.

*Positive lessons learned*: through the whole injury/surgery/rehabilitation process, patients expressed positive lessons, knowledge, insights, and changes at a personal level (i.e., being a stronger self). Greater knowledge of the body, the feeling of injury, the ability to cope with rehabilitation, and increased physical and mental strength are examples of reported personal growth. [Quotes #9, #10, #11, #12 (see Fig. 1)].

### Main category “Apprehensively peregrinating on an unknown road”

Thinking of the future, patients may experience fear, worry, and uncertainty. An uncertain future was compared with a path with no certain end, bearing life adaptations, and accepting that life after the injury would be different.

*Uncertainty about the future and osteoarthritis*: despite acceptable present knee functions, worry about future knee impairments was described. Specifically, the impact of osteoarthritis on knee-related quality of life was a matter of apprehension. Some patients reported missing healthcare information about the prevention of osteoarthritis.

*Being afraid for the knee and for what might happen*: patients were told that the injured knee should be trained throughout life. The idea of lifelong training to maintain acceptable knee function was not seen positively by the patients. The inability to take time off training was perceived as a stressor. Uncertainty about the future and fear of reinjury were described throughout the interviews.

*No wish for re-injury*: patients felt uncertain about being able to go through the same injury/surgery/rehabilitation process again and to find the motivation to complete rehabilitation. Patients also experienced uncertainty regarding whether the hard work during rehabilitation was worth the final outcome. Consequently, the patients expressed not wanting a second process of going through injury/surgery/rehabilitation. [Quotes #13, #14, #15 (see Fig. 1)].

## Discussion

The main results of this qualitative interview study comprised one theme, reflecting five main categories. Overall, some patients expressed their experiences of ACL injury more than 5 years after ACL reconstruction as a positive process, while others, due to persistent symptoms and unsatisfied expectations, expressed negative experiences and considered the ACL injury/reconstruction a life-changing event.

### Theme: to cope or not to cope, that is the question

The duality in experiences, where both positive and negative outcomes were experienced by patients more than 5 years after ACL reconstruction, can be reflected in the theme in our results: “to cope or not to cope, that is the question”. Two completely opposite sides of the same coin: patients can fully recover without thinking or worrying about their knee or experience persistent knee symptoms. The word “cope” in the theme is to be intended in its literal meaning: “to deal successfully with a difficult situation”. Nevertheless, the question of patients who might cope better (copers) than others (non-copers) after ACL injury has recently gained some light [14, 19] as it appears that the injury is characterized by variance in both symptoms and symptom resolutions. In published research on the topic, both copers and non-copers can be found: patients who succeed in a certain outcome (copers), for instance, returning to pre-injury level of sports, and patients who do not (non-copers), as research show up to 45% of patients who suffer an ACL injury do not resume competitive sport [2, 8]. As time passes from ACL injury, positive long-term experiences were displayed by some patients, while in contrast, persistent symptoms in the mid-to-long-term after ACL reconstruction were reported by other patients. Further research is important to better identify copers and non-copers early in rehabilitation.

### Main category: “Adapting life after knee symptoms: the past will not come back”

More than 5 years after ACL reconstruction, patients who perceived persistent knee symptoms when performing physical activities were forced to adapt their choices of physical activity to the knee. More than 20 years after ACL injury, patients appear to still report struggle with difficult decisions, such as whether to participate or not in different physical activities, and end up being less physically active [7]. To regain physical and mental balance after an ACL injury has been reported by patients as a difficult and long process, full of physical and mental challenges [12]. Assistance in accepting the life adaptations imposed by the knee might be warranted during rehabilitation after ACL injury/reconstruction.

### Main category: “An arduous and demanding rehabilitation: sailing against the wind”

Irrespective of whether patients experienced more or less physical and psychological limitations, rehabilitation after ACL reconstruction was long and demanding. In certain cases, patients expressed the time from injury to diagnosis as frustrating, since patients reported meeting incompetent, as they experienced them, healthcare professionals. For many patients, this meant that much time was spent worrying about what was wrong with their knee before receiving the diagnosis, contributing to the patients experiencing the process as negative. Patients meeting health care professionals who do not know how to assess and diagnose an ACL injury implies a need for further knowledge dissemination within the field of orthopedic injury assessment [17]. However, some patients saw the time from injury through diagnosis to surgery as an opportunity to rehabilitate before surgery to improve outcomes after surgery; therefore, the waiting time for surgery was not perceived as negative.

### Main category “Accepting what cannot be changed: biting the bullet”

For patients who did not cope well with ACL treatment and perceived persistent symptoms, the only reasonable choice was to try to accept the situation as it is and adapt to life after their injured knee. It is important to consider that symptoms such as knee joint swelling or pain after the knee demanding physical activity, considered acceptable at late-stage rehabilitation, might not resolve up to 5 years after ACL reconstruction. Therefore, physiotherapists should thoroughly assess [1, 18] patients after ACL reconstruction with standardized tests covering both aspects of physical as well as psychological function, prior to dismiss patients from rehabilitation to assess, evaluate, and possibly resolve all possible symptoms. The evaluation with standardized tests might allow clinicians to tailor interventions toward symptoms that need to be resolved before patients are discharged.

### Main category “Being satisfied with results: end of a chapter”

For patients who coped well, the ACL injury and reconstruction process was experienced as a closed chapter, and the patients were able to move on with their life without any substantial negative changes or adaptations. Instead, for these patients, the ACL injury/reconstruction process imposed positive changes, where patients reported to have learned more about their body, how to take care of the body, and learned important lessons about how to cope with difficult situations. Consequently, patients reported experiences of personal growth. Accordingly, in the treatment choice discussion between healthcare providers and patients after an ACL injury, our results suggest that for some patients, the injury and surgery process lead to positive outcomes, where patients feel psychologically strengthened.

### Main category “Apprehensively peregrinating on an unknown road”

Looking to the future, patients experienced uncertainty, not knowing if their knee would allow physical activity, and not knowing whether osteoarthritis would develop in the knee. Fear of re-injury was described by patients throughout the interviews. Fear of re-injury has been reported in patients treated with ACL reconstruction as a major hindrance for not returning to sport [13]. As fear of re-injury can still be present up to 5 years after ACL reconstruction, tailored interventions should be implemented. A concern regarding the need to be dependent on life-long knee training was expressed, with some patients stating that the inability to take periods off training felt like a prison. The clinical implications of this information stress the need to provide information about lifelong training at an early stage after injury.

### Methodological considerations

An ACL rupture and subsequent rehabilitation is a unique and highly subjective experience made of memories, sensations, feelings, impressions, and interaction with others. Qualitative content analysis is well-suited for analyzing complex experiences, as it can provide access to the subjective construction that each participant makes [9, 10]. As for the method choice, Graneheim and Lundman [10] proposed that data are found via an interaction between the researcher, participants, and analyzed text.

One crucial aspect of qualitative research is trustworthiness, which, according to the description of Graneheim and Lundman [10] can be further divided into credibility, dependability, and transferability. For credibility demographics of the included patients were described, and each involved research background and demographics were briefly described according to COREQs. To demonstrate the credibility of the analytical process, we have provided examples of the analytical process in Table 2, from codes to main categories. To ensure dependability, the interview guide was worked with before the study started and did not change afterward.

Qualitative research has limited generalizability, and transferability must be judged by the reader. In our sample of consecutively recruited patients, there were more women than men with a mean age of 30 years, which does not reflect the entire population of patients who suffer an ACL injury. However, the aim for this study was not to transfer the results to all patients who suffered an ACL injury, but rather to analyze how a certain subgroup might experience living with an ACL injured knee in the long term. Therefore, our results should be interpreted with caution.

The clinical relevance of this study is that it, from a patient perspective, confirms previous quantitative findings that some patients will cope (i.e., reach satisfactory outcomes) and some patients will not (i.e., not reaching satisfactory outcomes) after ACL reconstruction. The results stress the need for clinicians to transparently share information about possible long-term treatment outcomes, with patients treated with ACL reconstruction.

## Conclusions

More than 5 years after ACL reconstruction, patients might experience full symptom resolution and the ACL injury process as positive, or experience persistent symptoms and are forced to accept negative life-changing choices due to the injury. Further research is needed to better understand which patients will be copers and which will not.

## Supplementary Information

Below is the link to the electronic supplementary material.

Supplementary file1 (DOCX 14 KB)

## Data Availability

Data can be provided by corresponding author upon reasonable request.

## Abbreviations

ACL: Anterior cruciate ligament
COREQ: Consolidated criteria for reporting qualitative research
SRQR: Standards for reporting qualitative research
PT: Physiotherapist
