# Supplementary material for: Some, but not all, patients experience full symptom resolution and a positive rehabilitation process after ACL reconstruction: an interview study
Source: Knee Surg Sports Traumatol Arthrosc. 2022 Dec 9;31(7):2927–35. doi: 10.1007/s00167-022-07271-1 (PMC10276088; doi:10.1007/s00167-022-07271-1)
Supplement: Supplementary file 1 — Supplementary file1 (DOCX 14 KB) [file 167_2022_7271_MOESM1_ESM.docx]

**Appendix A**

Interview guide for ”Long-term experiences of patients after an ACL reconstruction” aiming to ”gain a deeper understating of the experiences of living with an altered knee in the long-term from patients after an ACL reconstruction”.

- Can you please describe how you injured your ACL?
- How are you phyiscally active today?
- (if active within a sport activity) What does your sport mean to you?
- Seen from a knee related perspective, how is your life today?
- What do you feel about your knee?
- How has the injury and rehabilitation process changed your life?
- Could you please tell me about your experience of rehabilitation?
- Which meaningful events have you experienced along the way?
- How do you feel it is to have an ACL injured knee today?
- Which consequences of the knee injury do you experience in your daily life?
- Please tell me about your knee-related function today?
- Do you experience any limitation related to your knee, and in case, which?
- What do you feel about the treatement you received, both surgery and rehabilitation?
- If you could go back in time, what would you change?
- How do you feel it is to have an ACL injured knee today?
- Do you have any proposition for improvement?
- Is there anything you would like to add?
